# Supplementary material for: Unveiling the Probiotic Potential of the Anaerobic Bacterium Cetobacterium sp. nov. C33 for Enhancing Nile Tilapia (Oreochromis niloticus) Cultures
Source: Microorganisms. 2023 Dec 5;11(12):2922. doi: 10.3390/microorganisms11122922 (PMC10745334; doi:10.3390/microorganisms11122922)
Supplement: Supplementary file 1 [file microorganisms-11-02922-s001.zip › microorganisms-2645832-supplementary.pdf]

microorganisms

MDPI

Table S1. Whole genome annotation of bacteria strain C33 with features of interest.

c	Subcategory	Subsystem	Role
Cofactors, Vitamins, Prosthetic Groups, Pigments	Biotin	Biotin biosynthesis	Dethiobiotin synthetase (EC 6.3.3.3)
			Biotin synthase (EC 2.8.1.6)
			Biotin operon repressor
			Adenosylmethionine-8-amino-7-oxononanoate aminotransferase (EC 2.6.1.62)
			8-amino-7-oxononanoate synthase (EC 2.3.1.47)
		Biotin biosynthesis Experimental	Dethiobiotin synthetase (EC 6.3.3.3)
			Biotin synthase (EC 2.8.1.6)
			Adenosylmethionine-8-amino-7-oxononanoate aminotransferase (EC 2.6.1.62)
			8-amino-7-oxononanoate synthase (EC 2.3.1.47)
	Cofactors, Vitamins, Prosthetic Groups, Pigments - no subcategory	Thiamin biosynthesis	Hydroxymethylpyrimidine ABC transporter, substrate-binding component
			Thiamin pyrophosphokinase (EC 2.7.6.2)
			Thiamine-monophosphate kinase (EC 2.7.4.16)
			1-deoxy-D-xylulose 5-phosphate synthase (EC 2.2.1.7)
			Thiamin ABC transporter, substrate-binding component
			Hydroxymethylpyrimidine ABC transporter, transmembrane component
			Thiamin-phosphate pyrophosphorylase (EC 2.5.1.3)
			Hydroxyethylthiazole kinase (EC 2.7.1.50)
	Tetrapyrroles	Cobalamin synthesis	L-threonine 3-O-phosphate decarboxylase (EC 4.1.1.81)
			Cobalt-precorrin-8x methylmutase (EC 5.4.1.2)
			Cobalt-precorrin-2 C20-methyltransferase (EC 2.1.1.130)
			Cobyric acid synthase (EC 6.3.5.10)
			Cobalamin biosynthesis protein CbiG
			Sirohydrochlorin cobaltochelatase CbiK (EC 4.99.1.3)
			Adenosylcobinamide kinase (EC 2.7.1.156)
			Cobalt-precorrin-6x reductase (EC 1.3.1.54)
			Adenosylcobinamide-phosphate guanylyltransferase (EC 2.7.7.62)
			Cobalt-precorrin-3b C17-methyltransferase
			Cobalt-precorrin-4 C11-methyltransferase (EC 2.1.1.133)
			Adenosylcobinamide-phosphate synthase (EC 6.3.1.10)
			Nicotinate-nucleotide--dimethylbenzimidazole phosphoribosyltransferase (EC 2.4.2.21)
			Alpha-ribazole-5'-phosphate phosphatase (EC 3.1.3.73)
			Cobalt-precorrin-6 synthase, anaerobic
			Cobalamin synthase (EC 2.7.8.26)
			Cobyrinic acid A,C-diamide synthase
		Heme and Siroheme Biosynthesis	Hypothetical radical SAM family enzyme, NOT coproporphyrinogen III oxidase, oxygen-independent
			Hypothetical radical SAM family enzyme in heat shock gene cluster, similarity with CPO of BS HemN-type
			Glutamyl-tRNA synthetase (EC 6.1.1.17)
			Uroporphyrinogen-III synthase (EC 4.2.1.75)
			Glutamate-1-semialdehyde aminotransferase (EC 5.4.3.8)
			Precorrin-2 oxidase (EC 1.3.1.76)
			Porphobilinogen synthase (EC 4.2.1.24)
			Glutamyl-tRNA reductase (EC 1.2.1.70)
			Porphobilinogen deaminase (EC 2.5.1.61)
			Uroporphyrinogen-III methyltransferase (EC 2.1.1.107)
			Sirohydrochlorin ferrochelatase (EC 4.99.1.4)
	Riboflavin, FMN, FAD		FMN adenylyltransferase (EC 2.7.7.2)
			FIG000859: hypothetical protein YebC

		Molybdopterin binding motif, CinA N-terminal domain
		Multi antimicrobial extrusion protein (Na(+)/drug antiporter), MATE family of MDR efflux pumps
		C-terminal domain of CinA type S
		6,7-dimethyl-8-ribityllumazine synthase (EC 2.5.1.78)
		Adenosine deaminase (EC 3.5.4.4), alternative form
		5-amino-6-(5-phosphoribosylamino)uracil reductase (EC 1.1.1.193)
	Riboflavin, FMN and FAD metabolism in plants	tRNA pseudouridine synthase B (EC 4.2.1.70)
		Riboflavin kinase (EC 2.7.1.26)
		GTP cyclohydrolase II (EC 3.5.4.25)
		tRNA (5-methylaminomethyl-2-thiouridylate)-methyltransferase (EC 2.1.1.61)
		Riboflavin synthase eubacterial/eukaryotic (EC 2.5.1.9)
		Diaminohydroxyphosphoribosylaminopyrimidine deaminase (EC 3.5.4.26)
		3,4-dihydroxy-2-butanone 4-phosphate synthase (EC 4.1.99.12)
	riboflavin to FAD	FMN adenylyltransferase (EC 2.7.7.2)
		Riboflavin synthase eubacterial/eukaryotic (EC 2.5.1.9)
		Riboflavin kinase (EC 2.7.1.26)
		3,4-dihydroxy-2-butanone 4-phosphate synthase (EC 4.1.99.12)
	Flavodoxin	Flavodoxin
		NAD(P)H oxidoreductase YRKL (EC 1.6.99.-)
		Flavodoxin 1
		Flavodoxin 2
	Riboflavin, FMN and FAD metabolism	FMN adenylyltransferase (EC 2.7.7.2)
		6,7-dimethyl-8-ribityllumazine synthase (EC 2.5.1.78)
		Adenosine deaminase (EC 3.5.4.4), alternative form
		5-amino-6-(5-phosphoribosylamino)uracil reductase (EC 1.1.1.193)
		Riboflavin kinase (EC 2.7.1.26)
		GTP cyclohydrolase II (EC 3.5.4.25)
		Riboflavin synthase eubacterial/eukaryotic (EC 2.5.1.9)
		Diaminohydroxyphosphoribosylaminopyrimidine deaminase (EC 3.5.4.26)
		3,4-dihydroxy-2-butanone 4-phosphate synthase (EC 4.1.99.12)
NAD and NADP	NAD and NADP cofactor biosynthesis global	Nicotinate phosphoribosyltransferase (EC 2.4.2.11)
		NAD kinase (EC 2.7.1.23)
		Glutamine amidotransferase chain of NAD synthetase
		C-terminal domain of CinA type S
		Nicotinate-nucleotide adenylyltransferase (EC 2.7.7.18)
		Nicotinamidase (EC 3.5.1.19)
		NAD synthetase (EC 6.3.1.5)
Folate and pterines	Folate biosynthesis cluster	GTP cyclohydrolase I (EC 3.5.4.16) type 1
		Cell division protein FtsH (EC 3.4.24.-)
		Dihydropteroate synthase (EC 2.5.1.15)
		Dihydroneopterin aldolase (EC 4.1.2.25)
		2-amino-4-hydroxy-6-hydroxymethyldihydropteridine pyrophosphokinase (EC 2.7.6.3)
		tRNA(Ile)-lysidine synthetase (EC 6.3.4.19)
		Hypoxanthine-guanine phosphoribosyltransferase (EC 2.4.2.8)
Coenzyme A	Coenzyme A Biosynthesis	Pantothenate:Na+ symporter (TC 2.A.21.1.1)
		Phosphopantetheine adenylyltransferase (EC 2.7.7.3)
		Ketol-acid reductoisomerase (EC 1.1.1.86)
		Phosphopantothenoylcysteine synthetase (EC 6.3.2.5)
		Phosphopantothenoylcysteine decarboxylase (EC 4.1.1.36)
		Dephospho-CoA kinase (EC 2.7.1.24)
		Pantothenate kinase type III, CoaX-like (EC 2.7.1.33)

Cell Wall and Capsule	Capsular and extracellular polysacchrides	dTDP-rhamnose synthesis	dTDP-4-dehydrorhamnose reductase (EC 1.1.1.133)
			Glucose-1-phosphate cytidylyltransferase (EC 2.7.7.33)
			dTDP-glucose 4,6-dehydratase (EC 4.2.1.46)
			dTDP-rhamnosyl transferase RfbF (EC 2.-.-.-)
			dTDP-4-dehydrorhamnose 3,5-epimerase (EC 5.1.3.13)
			Glucose-1-phosphate thymidylyltransferase (EC 2.7.7.24)
		Capsular heptose biosynthesis	Phosphoheptose isomerase (EC 5.3.1.-)
			GDP-mannose 4,6-dehydratase (EC 4.2.1.47)
			GDP-L-fucose synthetase (EC 1.1.1.271)
			dTDP-4-dehydrorhamnose 3,5-epimerase (EC 5.1.3.13)
			D-glycero-D-manno-heptose 1,7-bisphosphate phosphatase (EC 3.1.1.-)
		Polysaccharide deacetylases	Peptidoglycan N-acetylglucosamine deacetylase (EC 3.5.1.-)
			Polysaccharide deacetylase
		Rhamnose containing glycans	UDP-glucose 4-epimerase (EC 5.1.3.2)
			dTDP-4-dehydrorhamnose reductase (EC 1.1.1.133)
			capsular polysaccharide biosynthesis protein
			dTDP-glucose 4,6-dehydratase (EC 4.2.1.46)
			dTDP-4-dehydrorhamnose 3,5-epimerase (EC 5.1.3.13)
			Glucose-1-phosphate thymidylyltransferase (EC 2.7.7.24)
		Sialic Acid Metabolism	N-acetylneuraminate lyase (EC 4.1.3.3)
			Glucosamine-1-phosphate N-acetyltransferase (EC 2.3.1.157)
			Predicted sialic acid transporter
			TRAP-type transport system, large permease component, predicted N-acetylneuraminate transporter
			TRAP-type transport system, small permease component, predicted N-acetylneuraminate transporter
			N-acetylglucosamine-6-phosphate deacetylase (EC 3.5.1.25)
			Glucosamine--fructose-6-phosphate aminotransferase [isomerizing] (EC 2.6.1.16)
			Glucosamine-6-phosphate deaminase (EC 3.5.99.6)
			Sialic acid-induced transmembrane protein YjhT(NanM), possible mutarotase
			PTS system, N-acetylglucosamine-specific IIB component (EC 2.7.1.69)
			PTS system, N-acetylmuramic acid-specific IIB component (EC 2.7.1.69)
			N-acetylmannosamine-6-phosphate 2-epimerase (EC 5.1.3.9)
			N-acetylmannosamine kinase (EC 2.7.1.60)
			Phosphoglucosamine mutase (EC 5.4.2.10)
			N-acetylglucosamine-1-phosphate uridyltransferase (EC 2.7.7.23)
			TRAP-type transport system, periplasmic component, predicted N-acetylneuraminate-binding protein
			Sialic acid utilization regulator, RpiR family
	Gram-Negative cell wall components	LOS core oligosaccharide biosynthesis	Putative two-domain glycosyltransferase
			Phosphoheptose isomerase (EC 5.3.1.-)
			Beta-1,3-glucosyltransferase
			ADP-L-glycero-D-manno-heptose-6-epimerase (EC 5.1.3.20)
			D-glycero-D-manno-heptose 1,7-bisphosphate phosphatase (EC 3.1.1.-)
			Lipopolysaccharide core biosynthesis protein RfaY
			ADP-heptose synthase (EC 2.7.-.-)
			ADP-heptose--lipooligosaccharide heptosyltransferase II (EC 2.4.1.-)
			D-glycero-beta-D-manno-heptose 7-phosphate kinase
		Lipoprotein sorting system	Lipoprotein releasing system transmembrane protein LolC
			Lipoprotein releasing system ATP-binding protein LolD
		Murein Hydrolases	N-acetylmuramoyl-L-alanine amidase (EC 3.5.1.28)

			D-alanyl-D-alanine carboxypeptidase (EC 3.4.16.4)
			Soluble lytic murein transglycosylase precursor (EC 3.2.1.-)
			Muramoyltetrapeptide carboxypeptidase (EC 3.4.17.13)
	Cell Wall and Capsule - no subcategory	Peptidoglycan Biosynthesis	Glucosamine-1-phosphate N-acetyltransferase (EC 2.3.1.157)
			UDP-N-acetylmuramoylalanyl-D-glutamate--2,6-diaminopimelate ligase (EC 6.3.2.13)
			D-alanyl-D-alanine carboxypeptidase (EC 3.4.16.4)
			Cell division protein FtsI [Peptidoglycan synthetase] (EC 2.4.1.129)
			UDP-N-acetylenolpyruvoylglucosamine reductase (EC 1.1.1.158)
			Glutamine synthetase type III, GlnN (EC 6.3.1.2)
			UDP-N-acetylglucosamine 1-carboxyvinyltransferase (EC 2.5.1.7)
			UDP-N-acetylmuramoylalanine--D-glutamate ligase (EC 6.3.2.9)
			UDP-N-acetylglucosamine--N-acetylmuramyl-(pentapeptide) pyrophosphoryl-undecaprenol N-acetylglucosamine transferase (EC 2.4.1.227)
			Rod shape-determining protein RodA
			UDP-N-acetylmuramoylalanyl-D-glutamyl-2,6-diaminopimelate--D-alanyl-D-alanine ligase (EC 6.3.2.10)
			Glutamate racemase (EC 5.1.1.3)
			UDP-N-acetylmuramate--alanine ligase (EC 6.3.2.8)
			D-alanine--D-alanine ligase (EC 6.3.2.4)
			Phospho-N-acetylmuramoyl-pentapeptide-transferase (EC 2.7.8.13)
			N-acetylglucosamine-1-phosphate uridyltransferase (EC 2.7.7.23)
			Multimodular transpeptidase-transglycosylase (EC 2.4.1.129) (EC 3.4.-.-)
		UDP-N-acetylmuramate from Fructose-6-phosphate Biosynthesis	UDP-N-acetylenolpyruvoylglucosamine reductase (EC 1.1.1.158)
			Glucosamine-1-phosphate N-acetyltransferase (EC 2.3.1.157)
		UDP-N-acetylmuramate from Fructose-6-phosphate Biosynthesis	Glucosamine--fructose-6-phosphate aminotransferase [isomerizing] (EC 2.6.1.16)
			UDP-N-acetylglucosamine 1-carboxyvinyltransferase (EC 2.5.1.7)
			Phosphoglucosamine mutase (EC 5.4.2.10)
			N-acetylglucosamine-1-phosphate uridyltransferase (EC 2.7.7.23)
		YjeE	TsaB protein, required for threonylcarbamoyladenosine (t(6)A) formation in tRNA
			NAD(P)HX epimerase
			TsaE protein, required for threonylcarbamoyladenosine t(6)A37 formation in tRNA
			NAD(P)HX dehydratase
		Recycling of Peptidoglycan Amino Acids	N-acetylmuramoyl-L-alanine amidase (EC 3.5.1.28)
			Muramoyltetrapeptide carboxypeptidase (EC 3.4.17.13)
		Peptidoglycan biosynthesis--gjo	UDP-N-acetylmuramate--alanine ligase (EC 6.3.2.8)
			UDP-N-acetylmuramoylalanine--D-glutamate ligase (EC 6.3.2.9)
			UDP-N-acetylmuramoylalanyl-D-glutamate--2,6-diaminopimelate ligase (EC 6.3.2.13)
			D-alanine--D-alanine ligase (EC 6.3.2.4)
			UDP-N-acetylmuramoylalanyl-D-glutamyl-2,6-diaminopimelate--D-alanyl-D-alanine ligase (EC 6.3.2.10)
Virulence, Disease and Defense	Adhesion	Streptococcus pyogenes recombinatorial zone	Chaperonin (heat shock protein 33)
	Resistance to antibiotics and toxic compounds	Copper homeostasis	Copper-translocating P-type ATPase (EC 3.6.3.4)
		Cobalt-zinc-cadmium resistance	Cobalt-zinc-cadmium resistance protein
			Probable Co/Zn/Cd efflux system membrane fusion protein
			Transcriptional regulator, MerR family
		Resistance to fluoroquinolones	DNA gyrase subunit B (EC 5.99.1.3)
			DNA gyrase subunit A (EC 5.99.1.3)

		Arsenic resistance	Arsenical-resistance protein ACR3
			Arsenate reductase (EC 1.20.4.1)
		Beta-lactamase	Beta-lactamase (EC 3.5.2.6)
		Zinc resistance	Response regulator of zinc sigma-54-dependent two-component system
		Multidrug Resistance Efflux Pumps	Multi antimicrobial extrusion protein (Na(+)/drug antiporter), MATE family of MDR efflux pumps
			Acriflavin resistance protein
	Invasion and intracellular resistance	Mycobacterium virulence operon involved in protein synthesis (SSU ribosomal proteins)	SSU ribosomal protein S7p (S5e)
			Translation elongation factor G
			Translation elongation factor Tu
			SSU ribosomal protein S12p (S23e)
		Mycobacterium virulence operon involved in an unknown function with a Jag Protein and YidC and YidD	RNA-binding protein Jag
			Protein YidD
			Inner membrane protein translocase component YidC, short form OxaI-like
		Mycobacterium virulence operon involved in DNA transcription	DNA-directed RNA polymerase beta' subunit (EC 2.7.7.6)
			DNA-directed RNA polymerase beta subunit (EC 2.7.7.6)
		Mycobacterium virulence operon involved in protein synthesis (LSU ribosomal proteins)	LSU ribosomal protein L35p
			Translation initiation factor 3
			LSU ribosomal protein L20p
Potassium metabolism	Potassium metabolism - no subcategory	Potassium homeostasis	FKBP-type peptidyl-prolyl cis-trans isomerase SlyD (EC 5.2.1.8)
			Trk system potassium uptake protein TrkA
			Large-conductance mechanosensitive channel
			Kup system potassium uptake protein
Miscellaneous	Plant-Prokaryote DOE project	Conserved gene cluster possibly involved in RNA metabolism	Cysteinyl-tRNA synthetase (EC 6.1.1.16)
			RNA polymerase sporulation specific sigma factor SigH
			Serine acetyltransferase (EC 2.3.1.30)
			COG1939: Ribonuclease III family protein
	Miscellaneous - no subcategory	Phosphoglycerate mutase protein family	Alpha-ribazole-5'-phosphate phosphatase (EC 3.1.3.73)
			Carboxyl-terminal protease (EC 3.4.21.102)
			2,3-bisphosphoglycerate-independent phosphoglycerate mutase (EC 5.4.2.1)
		Muconate lactonizing enzyme family	L(+)-tartrate dehydratase beta subunit (EC 4.2.1.32)
			L(+)-tartrate dehydratase alpha subunit (EC 4.2.1.32)
		Broadly distributed proteins not in subsystems	YbbM seven transmembrane helix protein
			UPF0225 protein YchJ
			UPF0265 protein YeeX
Phages, Prophages, Transposable elements, Plasmids	Phages, Prophages	Phage DNA synthesis	Glutaredoxin
	Plasmid related functions	Rolling-circle replication	Replication initiation protein, topisomerase
Membrane Transport	ABC transporters	ABC transporter alkylphosphonate (TC 3.A.1.9.1)	Phosphonate ABC transporter ATP-binding protein (TC 3.A.1.9.1)
			Phosphonate ABC transporter phosphate-binding periplasmic component (TC 3.A.1.9.1)
		ABC transporter oligopeptide (TC 3.A.1.5.1)	Oligopeptide transport system permease protein OppC (TC 3.A.1.5.1)
			Oligopeptide transport system permease protein OppB (TC 3.A.1.5.1)
			Oligopeptide transport ATP-binding protein OppF (TC 3.A.1.5.1)
			Oligopeptide ABC transporter, periplasmic oligopeptide-binding protein OppA (TC 3.A.1.5.1)
		ABC transporter dipeptide (TC 3.A.1.5.2)	Dipeptide transport system permease protein DppB (TC 3.A.1.5.2)
			Dipeptide transport ATP-binding protein DppF (TC 3.A.1.5.2)

			Dipeptide transport ATP-binding protein DppD (TC 3.A.1.5.2)
			Dipeptide transport system permease protein DppC (TC 3.A.1.5.2)
			Dipeptide-binding ABC transporter, periplasmic substrate-binding component (TC 3.A.1.5.2)
	Protein translocation across cytoplasmic membrane	Bacterial signal recognition particle (SRP)	Signal recognition particle, subunit Ffh SRP54 (TC 3.A.5.1.1)
			Signal recognition particle associated protein
			Signal recognition particle receptor protein FtsY (=alpha subunit) (TC 3.A.5.1.1)
	Cation transporters	Magnesium transport	Magnesium and cobalt transport protein CorA
			Magnesium and cobalt efflux protein CorC
			Mg/Co/Ni transporter MgtE
			Mg(2+) transport ATPase, P-type (EC 3.6.3.2)
		Copper Transport System	Copper-translocating P-type ATPase (EC 3.6.3.4)
			Repressor CsoR of the copZA operon
	Uni- Sym- and Antiporters	NhaA, NhaD and Sodium-dependent phosphate transporters	Na+/H+ antiporter NhaD type
			Sodium-dependent phosphate transporter
	Membrane Transport - no subcategory	Choline Transport	Sodium-Choline Symporter
		ECF class transporters	Duplicated ATPase component MtsB of energizing module of methionine-regulated ECF transporter
			Transmembrane component MtsC of energizing module of methionine-regulated ECF transporter
			Substrate-specific component STY3230 of queuosine-regulated ECF transporter
			Substrate-specific component MtsA of methionine-regulated ECF transporter
		Ton and Tol transport systems	Ferric siderophore transport system, periplasmic binding protein TonB
			Outer membrane lipoprotein omp16 precursor
			MotA/TolQ/ExbB proton channel family protein
			TPR repeat containing exported protein
			Haemin uptake system outer membrane receptor
			Biopolymer transport protein ExbD/TolR
	TRAP transporters	TRAP Transporter collection	TRAP-type C4-dicarboxylate transport system, large permease component
			TRAP-type C4-dicarboxylate transport system, periplasmic component
Iron acquisition and metabolism	Iron acquisition and metabolism - no subcategory	Hemin transport system	Haemin uptake system permease protein
			Ferric siderophore transport system, periplasmic binding protein TonB
			Haemin uptake system outer membrane receptor
			Haemin uptake system ATP-binding protein
RNA Metabolism	RNA processing and modification	RNA pseudouridine syntheses	Ribosomal small subunit pseudouridine synthase A (EC 4.2.1.70)
			Ribosomal large subunit pseudouridine synthase D (EC 4.2.1.70)
			Ribosomal large subunit pseudouridine synthase C (EC 4.2.1.70)
			Ribosomal large subunit pseudouridine synthase B (EC 4.2.1.70)
			RNA pseudouridylate synthase BT0642
			tRNA pseudouridine synthase B (EC 4.2.1.70)
			tRNA pseudouridine synthase A (EC 4.2.1.70)
		tRNA nucleotidyltransferase	tRNA nucleotidyltransferase (EC 2.7.7.21) (EC 2.7.7.25)
		Methylthiotransferases	tRNA-t(6)A37 methylthiotransferase
			tRNA-i(6)A37 methylthiotransferase
			Ribosomal protein S12p Asp88 (E. coli) methylthiotransferase
		Ribonucleases in Bacillus	Ribonuclease HII (EC 3.1.26.4)
			Ribonuclease J2 (endoribonuclease in RNA processing)

	RNA processing and degradation, bacterial	Cytoplasmic axial filament protein CafA and Ribonuclease G (EC 3.1.4.-)
		3'-to-5' exoribonuclease RNase R
		FIG146085: 3'-to-5' oligoribonuclease A, Bacillus type
		Ribonuclease III (EC 3.1.26.3)
	RNA methylation	tRNA (guanosine(18)-2'-O)-methyltransferase (EC 2.1.1.34)
		tRNA (adenine37-N(6))-methyltransferase TrmN6 (EC 2.1.1.223)
		Ribosomal RNA small subunit methyltransferase E (EC 2.1.1.-)
		Ribosomal RNA large subunit methyltransferase F (EC 2.1.1.51)
		LSU m5C1962 methyltransferase RlmI
		SSU rRNA (adenine(1518)-N(6)/adenine(1519)-N(6))-dimethyl-transferase (EC 2.1.1.182)
		23S rRNA (guanosine-2'-O-) -methyltransferase rlmB (EC 2.1.1.-)
		tRNA (guanine46-N7-)-methyltransferase (EC 2.1.1.33)
		Ribosomal RNA large subunit methyltransferase N (EC 2.1.1.-)
		tRNA (Guanine37-N1) -methyltransferase (EC 2.1.1.31)
		tRNA (5-methylaminomethyl-2-thiouridylate)-methyltransferase (EC 2.1.1.61)
		tRNA (cytidine(34)-2'-O)-methyltransferase (EC 2.1.1.207)
		LSU m3Psi1915 methyltransferase RlmH
		rRNA small subunit 7-methylguanosine (m7G) methyltransferase GidB
		tRNA-specific 2-thiouridylase MnmA
	ATP-dependent RNA helicases, bacterial	ATP-dependent RNA helicase RhlE
		ATP-dependent RNA helicase BA2475
	16S rRNA modification within P site of ribosome	Cell division protein FtsI [Peptidoglycan synthetase] (EC 2.4.1.129)
		rRNA small subunit methyltransferase H
		rRNA small subunit methyltransferase I
	mnm5U34 biosynthesis bacteria	GTPase and tRNA-U34 5-formylation enzyme TrmE
		tRNA uridine 5-carboxymethylaminomethyl modification enzyme GidA
		tRNA (5-methylaminomethyl-2-thiouridylate)-methyltransferase (EC 2.1.1.61)
		Cysteine desulfurase (EC 2.8.1.7)
	Queuosine-Archaeosine Biosynthesis	preQ1-regulated inosine-uridine nucleoside hydrolase (EC 3.2.2.1)
		Peptidyl-prolyl cis-trans isomerase (EC 5.2.1.8)
		Inosine-uridine preferring nucleoside hydrolase (EC 3.2.2.1)
		Putative preQ0 transporter
		S-adenosylmethionine:tRNA ribosyltransferase-isomerase (EC 5.-.-.-)
		Permease of the drug/metabolite transporter (DMT) superfamily
		GTP cyclohydrolase I (EC 3.5.4.16) type 1
		tRNA-guanine transglycosylase (EC 2.4.2.29)
		Substrate-specific component STY3230 of queuosine-regulated ECF transporter
	Ribonuclease H	Ribonuclease HII (EC 3.1.26.4)
	tRNA processing	Ribonuclease P protein component (EC 3.1.26.5)
		tRNA-i(6)A37 methylthiotransferase
		tRNA dimethylallyltransferase (EC 2.5.1.75)
		tRNA(Ile)-lysidine synthetase (EC 6.3.4.19)
		Ribonuclease PH (EC 2.7.7.56)
		tRNA pseudouridine synthase B (EC 4.2.1.70)
		tRNA pseudouridine synthase A (EC 4.2.1.70)
Transcription	Transcription initiation, bacterial sigma factors	RNA polymerase sigma-54 factor RpoN
		RNA polymerase sigma factor RpoD
		RNA polymerase sporulation specific sigma factor SigH
		DNA-directed RNA polymerase alpha subunit (EC 2.7.7.6)

		RNA polymerase bacterial	DNA-directed RNA polymerase beta' subunit (EC 2.7.7.6)
			DNA-directed RNA polymerase omega subunit (EC 2.7.7.6)
			DNA-directed RNA polymerase beta subunit (EC 2.7.7.6)
		Transcription factors bacterial	Transcription termination protein NusB
			Transcription accessory protein (S1 RNA-binding domain)
			Transcription termination factor Rho
			Transcription antitermination protein NusG
			Transcription-repair coupling factor
			ribosomal protein L7Ae family protein
			COG2740: Predicted nucleic-acid-binding protein implicated in transcription termination
			FIG000325: clustered with transcription termination protein NusA
			Transcription termination protein NusA
		Rrf2 family transcriptional regulators	Rrf2 family transcriptional regulator
	RNA Metabolism - no subcategory	Group II intron-associated genes	Retron-type RNA-directed DNA polymerase (EC 2.7.7.49)
Nucleosides and Nucleotides	Pyrimidines	pyrimidine conversions	Nucleoside diphosphate kinase (EC 2.7.4.6)
			Uridine kinase (EC 2.7.1.48) [C1]
			Thymidylate kinase (EC 2.7.4.9)
			Cytosine deaminase (EC 3.5.4.1)
			Thioredoxin reductase (EC 1.8.1.9)
			Pyrimidine-nucleoside phosphorylase (EC 2.4.2.2)
			Cytidylate kinase (EC 2.7.4.25)
			Thymidine kinase (EC 2.7.1.21)
			Bis(5'-nucleosyl)-tetraphosphatase (asymmetrical) (EC 3.6.1.17)
			Purine nucleoside phosphorylase (EC 2.4.2.1)
			5'-nucleotidase (EC 3.1.3.5)
			Cytidine deaminase (EC 3.5.4.5)
			Uridine kinase (EC 2.7.1.48)
			Uracil phosphoribosyltransferase (EC 2.4.2.9)
			2',3'-cyclic-nucleotide 2'-phosphodiesterase (EC 3.1.4.16)
			CTP synthase (EC 6.3.4.2)
		De Novo Pyrimidine Synthesis	Uracil permease
			Orotidine 5'-phosphate decarboxylase (EC 4.1.1.23)
			Dihydroorotate dehydrogenase electron transfer subunit (EC 1.3.3.1)
			Aspartate carbamoyltransferase (EC 2.1.3.2)
			Dihydroorotate dehydrogenase, catalytic subunit (EC 1.3.3.1)
			Carbamoyl-phosphate synthase small chain (EC 6.3.5.5)
			Orotate phosphoribosyltransferase (EC 2.4.2.10)
			Carbamoyl-phosphate synthase large chain (EC 6.3.5.5)
			Uracil phosphoribosyltransferase (EC 2.4.2.9)
			Dihydroorotase (EC 3.5.2.3)
	Purines	De Novo Purine Biosynthesis	Phosphoribosylamine--glycine ligase (EC 6.3.4.13)
			IMP cyclohydrolase (EC 3.5.4.10)
			Amidophosphoribosyltransferase (EC 2.4.2.14)
			Ribose-phosphate pyrophosphokinase (EC 2.7.6.1)
			Phosphoribosylaminoimidazole carboxylase catalytic subunit (EC 4.1.1.21)
			Phosphoribosylglycinamide formyltransferase (EC 2.1.2.2)
			Phosphoribosylaminoimidazole-succinocarboxamide synthase (EC 6.3.2.6)
			Adenylosuccinate lyase (EC 4.3.2.2)
			Phosphoribosylformylglycinamidine cyclo-ligase (EC 6.3.3.1)

			Phosphoribosylaminoimidazolecarboxamide formyltransferase (EC 2.1.2.3)
			Phosphoribosylformylglycinamidine synthase, synthetase subunit (EC 6.3.5.3)
			Phosphoribosylaminoimidazole carboxylase ATPase subunit (EC 4.1.1.21)
			Phosphoribosylformylglycinamidine synthase, glutamine amidotransferase subunit (EC 6.3.5.3)
		Xanthine Metabolism in Bacteria	Xanthine phosphoribosyltransferase (EC 2.4.2.22)
		Purine Utilization	Xanthine/uracil/thiamine/ascorbate permease family protein
			Xanthine and CO dehydrogenases maturation factor, XdhC/CoxF family
			Uracil-xanthine permease
		Purine conversions	preQ1-regulated inosine-uridine nucleoside hydrolase (EC 3.2.2.1)
			GMP synthase [glutamine-hydrolyzing] (EC 6.3.5.2)
			Adenylosuccinate synthetase (EC 6.3.4.4)
			Inosine-5'-monophosphate dehydrogenase (EC 1.1.1.205)
			Hypoxanthine-guanine phosphoribosyltransferase (EC 2.4.2.8)
			Possible hypoxanthine oxidase XdhD (EC 1.-.-.-)
			Adenylosuccinate lyase (EC 4.3.2.2)
			Polyphosphate kinase (EC 2.7.4.1)
			Deoxyadenosine kinase (EC 2.7.1.76)
			Adenine phosphoribosyltransferase (EC 2.4.2.7)
			2',3'-cyclic-nucleotide 2'-phosphodiesterase (EC 3.1.4.16)
			Adenylate kinase (EC 2.7.4.3)
			Inosine-uridine preferring nucleoside hydrolase (EC 3.2.2.1)
			Nucleoside diphosphate kinase (EC 2.7.4.6)
			Deoxyguanosine kinase (EC 2.7.1.113)
			Adenine deaminase (EC 3.5.4.2)
			Purine nucleoside phosphorylase (EC 2.4.2.1)
			5'-nucleotidase (EC 3.1.3.5)
			Xanthine phosphoribosyltransferase (EC 2.4.2.22)
			Adenosine deaminase (EC 3.5.4.4)
			Guanylate kinase (EC 2.7.4.8)
	Nucleosides and Nucleotides - no subcategory	Ribonucleotide reduction	Ribonucleotide reductase of class III (anaerobic), activating protein (EC 1.97.1.4)
			Ribonucleotide reductase of class III (anaerobic), large subunit (EC 1.17.4.2)
			Ribonucleotide reductase of class Ia (aerobic), beta subunit (EC 1.17.4.1)
			Ribonucleotide reductase of class Ia (aerobic), alpha subunit (EC 1.17.4.1)
			Ribonucleotide reductase transcriptional regulator NrdR
		Pseudouridine catabolism	Pseudouridine 5'-phosphate glycosidase
			Pseudouridine kinase (EC 2.7.1.83)
		Adenosyl nucleosidases	S-adenosylhomocysteine nucleosidase (EC 3.2.2.9)
			Purine nucleoside phosphorylase (EC 2.4.2.1)
			5'-methylthioadenosine nucleosidase (EC 3.2.2.16)
	Detoxification	Nucleoside triphosphate pyrophosphohydrolase MazG	Nucleoside triphosphate pyrophosphohydrolase MazG (EC 3.6.1.8)
		Nudix proteins (nucleoside triphosphate hydrolases)	Deoxyuridine 5'-triphosphate nucleotidohydrolase (EC 3.6.1.23)
			Hypothetical nudix hydrolase YeaB
			Deoxyuridine 5'-triphosphate nucleotidohydrolase (EC 3.6.1.23)

		Housecleaning nucleoside triphosphate pyrophosphatases	Nucleoside 5-triphosphatase RdgB (dHAPTP, dITP, XTP-specific) (EC 3.6.1.15)
			5'-nucleotidase YjjG (EC 3.1.3.5)
Protein Metabolism	Protein folding	GroEL GroES	Heat shock protein GrpE
			Heat-inducible transcription repressor HrcA
			Chaperone protein DnaK
			Chaperone protein DnaJ
			Heat shock protein 60 family chaperone GroEL
		Protein chaperones	Heat shock protein GrpE
			Chaperone protein DnaK
			Chaperone protein DnaJ
			Chaperone protein HtpG
			ClpB protein
		Periplasmic disulfide interchange	Cytochrome c-type biogenesis protein CcdA (DsbD analog)
		Peptidyl-prolyl cis-trans isomerase	Survival protein SurA precursor (Peptidyl-prolyl cis-trans isomerase SurA) (EC 5.2.1.8)
			FKBP-type peptidyl-prolyl cis-trans isomerase SlyD (EC 5.2.1.8)
	Selenoproteins	Selenocysteine metabolism	selenocysteine-containing
			L-seryl-tRNA(Sec) selenium transferase (EC 2.9.1.1)
			Selenophosphate-dependent tRNA 2-selenouridine synthase
			Selenocysteine-specific translation elongation factor
			Selenide,water dikinase (EC 2.7.9.3)
	Protein biosynthesis	tRNA aminoacylation, Val	Valyl-tRNA synthetase (EC 6.1.1.9)
		tRNA aminoacylation, Met	Methionyl-tRNA synthetase (EC 6.1.1.10)
		tRNA aminoacylation, Ile	Isoleucyl-tRNA synthetase (EC 6.1.1.5)
		tRNA aminoacylation, Arg	Arginyl-tRNA synthetase (EC 6.1.1.19)
		Translation initiation factors bacterial	Translation initiation factor 1
			Methionyl-tRNA formyltransferase (EC 2.1.2.9)
			Translation initiation factor 3
			Translation initiation factor 2
			Ribosome-binding factor A
		tRNA aminoacylation, Gly	Glycyl-tRNA synthetase alpha chain (EC 6.1.1.14)
			Glycyl-tRNA synthetase beta chain (EC 6.1.1.14)
		tRNA aminoacylation, Ala	Alanyl-tRNA synthetase family protein
			Alanyl-tRNA synthetase (EC 6.1.1.7)
		tRNA aminoacylation, Trp	Tryptophanyl-tRNA synthetase (EC 6.1.1.2)
		Ribosome biogenesis bacterial	16S rRNA processing protein RimM
			TsaB protein, required for threonylcarbamoyladenosine (t(6)A) formation in tRNA
			Ribosomal large subunit pseudouridine synthase D (EC 4.2.1.70)
			Ribosomal large subunit pseudouridine synthase C (EC 4.2.1.70)
			LSU m5C1962 methyltransferase RlmI
			SSU rRNA (adenine(1518)-N(6)/adenine(1519)-N(6))-dimethyltransferase (EC 2.1.1.182)
			tRNA (Guanine37-N1) -methyltransferase (EC 2.1.1.31)
			Ribosomal-protein-S18p-alanine acetyltransferase (EC 2.3.1.-)
			Ribonuclease M5 (EC 3.1.26.8)
			LSU m3Psi1915 methyltransferase RlmH
			Ribosomal protein L11 methyltransferase (EC 2.1.1.-)
		Programmed frameshift	programmed frameshift-containing

	Peptide chain release factor 2
tRNA aminoacylation, Cys	Cysteinyl-tRNA synthetase (EC 6.1.1.16)
Translation termination factors bacterial	Protein-N(5)-glutamine methyltransferase PrmC, methylates polypeptide chain release factors RF1 and RF2
	Ribosome recycling factor
	Peptidyl-tRNA hydrolase (EC 3.1.1.29)
	Peptide deformylase (EC 3.5.1.88)
	tmRNA-binding protein SmpB
	Peptide chain release factor 1
	Methionine aminopeptidase (EC 3.4.11.18)
	Peptide chain release factor 2
Universal GTPases	Signal recognition particle, subunit Ffh SRP54 (TC 3.A.5.1.1)
	GTP-binding protein EngA
	GTP-binding protein YqeH, required for biogenesis of 30S ribosome subunit
	GTP-binding protein HflX
	Signal recognition particle receptor protein FtsY (=alpha subunit) (TC 3.A.5.1.1)
	50S ribosomal subunit maturation GTPase RbgA (B. subtilis YlqF)
	GTP-binding protein TypA/BipA
	Translation elongation factor LepA
	Translation initiation factor 2
	Ribosome small subunit-stimulated GTPase EngC
	Probable GTPase related to EngC
	Translation elongation factor G
	GTP-binding protein Era
	GTPase and tRNA-U34 5-formylation enzyme TrmE
	GTP-binding and nucleic acid-binding protein YchF
	Translation elongation factor Tu
	GTP-binding protein Obg
	GTP-binding protein EngB
tRNA aminoacylation, His	Histidyl-tRNA synthetase (EC 6.1.1.21)
tRNA aminoacylation, Asp and Asn	Aspartyl-tRNA(Asn) amidotransferase subunit C (EC 6.3.5.6)
	Aspartyl-tRNA(Asn) amidotransferase subunit B (EC 6.3.5.6)
tRNA aminoacylation, Asp and Asn	Aspartyl-tRNA synthetase (EC 6.1.1.12)
	Asparaginyl-tRNA synthetase (EC 6.1.1.22)
	Aspartyl-tRNA(Asn) amidotransferase subunit A (EC 6.3.5.6)
Translation elongation factors bacterial	Translation elongation factor G
	Translation elongation factor LepA
	Translation elongation factor Tu
	Translation elongation factor Ts
	Translation elongation factor P
	Translation elongation factor G-related protein
tRNA aminoacylation, Lys	Lysyl-tRNA synthetase (class II) (EC 6.1.1.6)
tRNA aminoacylation, Thr	Threonyl-tRNA synthetase (EC 6.1.1.3)
tRNA aminoacylation, Pro	Prolyl-tRNA synthetase (EC 6.1.1.15), bacterial type
Translation elongation factor G family	Translation elongation factor G
	Tetracycline resistance protein TetP
	Translation elongation factor G-related protein
	Glutamyl-tRNA(Gln) amidotransferase subunit A (EC 6.3.5.7)

		tRNA aminoacylation, Glu and Gln	Glutamyl-tRNA synthetase (EC 6.1.1.17)
			Glutamyl-tRNA(Gln) amidotransferase subunit B (EC 6.3.5.7)
			Glutamyl-tRNA(Gln) synthetase (EC 6.1.1.24)
			Glutamyl-tRNA(Gln) amidotransferase subunit C (EC 6.3.5.7)
		tRNA aminoacylation, Ser	Seryl-tRNA synthetase (EC 6.1.1.11)
		tRNA aminoacylation, Tyr	Tyrosyl-tRNA synthetase (EC 6.1.1.1)
		tRNA aminoacylation, Leu	Leucyl-tRNA synthetase (EC 6.1.1.4)
		Glycyl-tRNA synthetase	Glycyl-tRNA synthetase alpha chain (EC 6.1.1.14)
			Glycyl-tRNA synthetase beta chain (EC 6.1.1.14)
		tRNA aminoacylation, Phe	Phenylalanyl-tRNA synthetase alpha chain (EC 6.1.1.20)
			Phenylalanyl-tRNA synthetase beta chain (EC 6.1.1.20)
	Protein processing and modification	Lipoprotein Biosynthesis	Lipoprotein signal peptidase (EC 3.4.23.36)
			Prolipoprotein diacylglyceryl transferase (EC 2.4.99.-)
		Signal peptidase	Signal peptidase I (EC 3.4.21.89)
			Lipoprotein signal peptidase (EC 3.4.23.36)
		Ribosomal protein S12p Asp methylthiotransfer-ase	Ribosomal protein S12p Asp88 (E. coli) methylthiotransferase
			SSU ribosomal protein S12p (S23e)
		G3E family of P-loop GTPases (metallocenter biosynthesis)	Urease accessory protein UreD
			FKBP-type peptidyl-prolyl cis-trans isomerase SlyD (EC 5.2.1.8)
			Urease accessory protein UreF
			Urease beta subunit (EC 3.5.1.5)
			Urease accessory protein UreG
			Urease gamma subunit (EC 3.5.1.5)
			Urease alpha subunit (EC 3.5.1.5)
		Peptide methionine sul-foxide reductase	Peptide methionine sulfoxide reductase MsrA (EC 1.8.4.11)
			Peptide methionine sulfoxide reductase MsrB (EC 1.8.4.12)
	Protein degradation	Putative TldE-TldD pro-teolytic complex	TldE protein, part of TldE/TldD proteolytic complex
			TldD protein, part of TldE/TldD proteolytic complex
		Aminopeptidases (EC 3.4.11.-)	Xaa-Pro aminopeptidase (EC 3.4.11.9)
			Cytosol aminopeptidase PepA (EC 3.4.11.1)
		Protein degradation	Deblocking aminopeptidase (EC 3.4.11.-)
			Aminopeptidase YpdF (MP-, MA-, MS-, AP-, NP- specific)
		Metallocarboxypeptida-ses (EC 3.4.17.-)	D-alanyl-D-alanine carboxypeptidase (EC 3.4.16.4)
			Muramoyltetrapeptide carboxypeptidase (EC 3.4.17.13)
			Thermostable carboxypeptidase 1 (EC 3.4.17.19)
		Proteolysis in bacteria, ATP-dependent	DNA repair protein RadA
			ATP-dependent Clp protease ATP-binding subunit ClpX
			ATP-dependent Clp protease proteolytic subunit (EC 3.4.21.92)
			ClpB protein
			ATP-dependent protease La (EC 3.4.21.53) Type I
		Omega peptidases (EC 3.4.19.-)	Pyrrolidone-carboxylate peptidase (EC 3.4.19.3)
Cell Division and Cell Cycle	Cell Division and Cell Cycle - no sub-category	YgjD and YeaZ	TsaD/Kae1/Qri7 protein, required for threonylcarbamoyladenosine t(6)A37 formation in tRNA
			TsaB protein, required for threonylcarbamoyladenosine (t(6)A) formation in tRNA
		Macromolecular synthe-sis operon	TsaD/Kae1/Qri7 protein, required for threonylcarbamoyladenosine t(6)A37 formation in tRNA
			RNA polymerase sigma factor RpoD
			DNA primase (EC 2.7.7.-)
			Carbamoyl-phosphate synthase small chain (EC 6.3.5.5)
			Carbamoyl-phosphate synthase large chain (EC 6.3.5.5)

		Bacterial Cytoskeleton	Septum site-determining protein MinC
			Cell division protein FtsI [Peptidoglycan synthetase] (EC 2.4.1.129)
			Rod shape-determining protein RodA
			Cell division protein FtsW
			Cell division protein FtsZ (EC 3.4.24.-)
			Cell division protein FtsA
			Rod shape-determining protein MreB
			Rod shape-determining protein MreC
			Septum formation protein Maf
			Septum site-determining protein MinD
			Cell division topological specificity factor MinE
			Chromosome (plasmid) partitioning protein ParA
Regulation and Cell signaling	Regulation and Cell signaling - no subcategory	cAMP signaling in bacteria	ElaA protein
			ATP-dependent Clp protease proteolytic subunit (EC 3.4.21.92)
			Adenylate cyclase (EC 4.6.1.1)
			Adenine phosphoribosyltransferase (EC 2.4.2.7)
			3',5'-cyclic-nucleotide phosphodiesterase (EC 3.1.4.17)
		HPr catabolite repression system	Catabolite control protein A
			HPr kinase/phosphorylase (EC 2.7.1.-) (EC 2.7.4.-)
		Stringent Response, (p)ppGpp metabolism	GTP pyrophosphokinase (EC 2.7.6.5), (p)ppGpp synthetase I
	Programmed Cell Death and Toxin-antitoxin Systems	Toxin-antitoxin replicon stabilization systems	DNA-damage-inducible protein J
Secondary Metabolism	Plant Alkaloids	Alkaloid biosynthesis from L-lysine	Oxalyl-CoA decarboxylase (EC 4.1.1.8)
DNA Metabolism	DNA repair	Uracil-DNA glycosylase	Uracil-DNA glycosylase, family 1
		DNA repair, bacterial MutL-MutS system	Recombination inhibitory protein MutS2
			DNA mismatch repair protein MutS
			DNA mismatch repair protein MutL
		DNA repair, UvrABC system	Excinuclease ABC subunit C
			Excinuclease ABC subunit A
			Excinuclease ABC subunit B
		DNA repair, bacterial photolyase	Deoxyribodipyrimidine photolyase, type II (EC 4.1.99.3)
		DNA repair, bacterial DinG and relatives	DinG family ATP-dependent helicase YoaA
		DNA repair system including RecA, MutS and a hypothetical protein	DNA mismatch repair protein MutS
			RecA protein
		DNA repair, bacterial	Exodeoxyribonuclease VII large subunit (EC 3.1.11.6)
			DNA repair protein RadA
			Exonuclease SbcD
			DNA repair protein RecN
			DNA repair protein RadC
			DNA polymerase IV (EC 2.7.7.7)
			Exonuclease SbcC
			DNA-damage-inducible protein J
			Single-stranded DNA-binding protein
			Methylated-DNA--protein-cysteine methyltransferase (EC 2.1.1.63)
			Endonuclease IV (EC 3.1.21.2)
			RecA protein
			Exodeoxyribonuclease III (EC 3.1.11.2)
			Exodeoxyribonuclease VII small subunit (EC 3.1.11.6)
			ATP-dependent DNA helicase RecQ

		DNA repair, bacterial RecFOR pathway	Single-stranded-DNA-specific exonuclease RecJ (EC 3.1.-.-)
			Single-stranded DNA-binding protein
			Recombination protein RecR
			RecA protein
			DNA recombination and repair protein RecO
			DNA recombination and repair protein RecF
		DNA Repair Base Excision	Endonuclease III (EC 4.2.99.18)
			Single-stranded-DNA-specific exonuclease RecJ (EC 3.1.-.-)
			Uracil-DNA glycosylase, family 1
			DNA-3-methyladenine glycosylase (EC 3.2.2.20)
			DNA polymerase I (EC 2.7.7.7)
			DNA ligase (EC 6.5.1.2)
		DNA repair, bacterial UvrD and related helicases	ATP-dependent DNA helicase UvrD/PcrA
	CRISPs	CRISPRs	CRISPR-associated protein, Csn1 family
			CRISPR-associated protein Cas1
			CRISPR-associated protein, Csm1 family
	DNA Metabolism - no subcategory	Type I Restriction-Modification	Type I restriction-modification system, specificity subunit S (EC 3.1.21.3)
			Type I restriction-modification system, restriction subunit R (EC 3.1.21.3)
			Type I restriction-modification system, DNA-methyltransferase subunit M (EC 2.1.1.72)
		Restriction-Modification System	Type I restriction-modification system, specificity subunit S (EC 3.1.21.3)
			Type I restriction-modification system, restriction subunit R (EC 3.1.21.3)
			Type I restriction-modification system, DNA-methyltransferase subunit M (EC 2.1.1.72)
			Putative predicted metal-dependent hydrolase
		YcfH	Putative deoxyribonuclease YcfH
		DNA structural proteins, bacterial	Chromosome partition protein smc
			DNA-binding protein HU
	DNA replication	DNA topoisomerases, Type I, ATP-independent	DNA topoisomerase III (EC 5.99.1.2)
			DNA topoisomerase I (EC 5.99.1.2)
		DNA topoisomerases, Type II, ATP-dependent	DNA gyrase subunit B (EC 5.99.1.3)
			DNA gyrase subunit A (EC 5.99.1.3)
	DNA recombination	RuvABC plus a hypothetical	FIG000859: hypothetical protein YebC
			Crossover junction endodeoxyribonuclease RuvC (EC 3.1.22.4)
			Holliday junction DNA helicase RuvA
			Holliday junction DNA helicase RuvB
	DNA uptake, competence	DNA processing cluster	FIG000557: hypothetical protein co-occurring with RecR
			Recombination protein RecR
			DNA topoisomerase III (EC 5.99.1.2)
			DNA polymerase III subunits gamma and tau (EC 2.7.7.7)
Fatty Acids, Lipids, and Isoprenoids	Phospholipids	Cardiolipin synthesis	Cardiolipin synthetase (EC 2.7.8.-)
		Glycerolipid and Glycerophospholipid Metabolism in Bacteria	Alcohol dehydrogenase (EC 1.1.1.1)
			Acyl-phosphate:glycerol-3-phosphate O-acyltransferase PlsY
			Phosphatidylglycerophosphatase A (EC 3.1.3.27)
			CDP-diacylglycerol--serine O-phosphatidyltransferase (EC 2.7.8.8)
			Glycerol-3-phosphate dehydrogenase (EC 1.1.5.3)
			Glycerol-3-phosphate dehydrogenase [NAD(P)+] (EC 1.1.1.94)
			Glycerol kinase (EC 2.7.1.30)

			Phosphatidylserine decarboxylase (EC 4.1.1.65)
			Aldehyde dehydrogenase (EC 1.2.1.3)
			1-acyl-sn-glycerol-3-phosphate acyltransferase (EC 2.3.1.51)
			Diacylglycerol kinase (EC 2.7.1.107)
			CDP-diacylglycerol--glycerol-3-phosphate 3-phosphatidyltransferase (EC 2.7.8.5)
			Cardiolipin synthetase (EC 2.7.8.-)
			Dihydroxyacetone kinase family protein
			Acyl carrier protein
			Phosphatidate cytidylyltransferase (EC 2.7.7.41)
			Phosphate:acyl-ACP acyltransferase PlsX
			Glycerate kinase (EC 2.7.1.31)
	Triacylglycerols	Triacylglycerol metabolism	Monoglyceride lipase (EC 3.1.1.23)
			Lysophospholipase (EC 3.1.1.5)
	Fatty acids	Fatty Acid Biosynthesis FASII	3-oxoacyl-[acyl-carrier protein] reductase (EC 1.1.1.100)
			Acetyl-coenzyme A carboxyl transferase alpha chain (EC 6.4.1.2)
			Holo-[acyl-carrier protein] synthase (EC 2.7.8.7)
			Biotin carboxyl carrier protein of acetyl-CoA carboxylase
			Biotin carboxylase of acetyl-CoA carboxylase (EC 6.3.4.14)
			Acetyl-coenzyme A carboxyl transferase beta chain (EC 6.4.1.2)
			Acyl carrier protein
			Malonyl CoA-acyl carrier protein transacylase (EC 2.3.1.39)
			3-hydroxyacyl-[acyl-carrier-protein] dehydratase, FabZ form (EC 4.2.1.59)
			Enoyl-[acyl-carrier-protein] reductase [FMN] (EC 1.3.1.9)
	Isoprenoids	Isoprenoinds for Quinones	Undecaprenyl diphosphate synthase (EC 2.5.1.31)
			Heptaprenyl diphosphate synthase component I (EC 2.5.1.30)
		Isoprenoid Biosynthesis	1-deoxy-D-xylulose 5-phosphate reductoisomerase (EC 1.1.1.267)
			Undecaprenyl diphosphate synthase (EC 2.5.1.31)
			4-hydroxy-3-methylbut-2-enyl diphosphate reductase (EC 1.17.1.2)
			1-hydroxy-2-methyl-2-(E)-butenyl 4-diphosphate synthase (EC 1.17.7.1)
			4-diphosphocytidyl-2-C-methyl-D-erythritol kinase (EC 2.7.1.148)
			2-C-methyl-D-erythritol 2,4-cyclodiphosphate synthase (EC 4.6.1.12)
			2-C-methyl-D-erythritol 4-phosphate cytidylyltransferase (EC 2.7.7.60)
			1-deoxy-D-xylulose 5-phosphate synthase (EC 2.2.1.7)
		Nonmevalonate Branch of Isoprenoid Biosynthesis	1-deoxy-D-xylulose 5-phosphate reductoisomerase (EC 1.1.1.267)
			4-hydroxy-3-methylbut-2-enyl diphosphate reductase (EC 1.17.1.2)
			1-hydroxy-2-methyl-2-(E)-butenyl 4-diphosphate synthase (EC 1.17.7.1)
			4-diphosphocytidyl-2-C-methyl-D-erythritol kinase (EC 2.7.1.148)
			2-C-methyl-D-erythritol 2,4-cyclodiphosphate synthase (EC 4.6.1.12)
			2-C-methyl-D-erythritol 4-phosphate cytidylyltransferase (EC 2.7.7.60)
			1-deoxy-D-xylulose 5-phosphate synthase (EC 2.2.1.7)
Nitrogen Metabolism	Nitrogen Metabolism - no subcategory	Nitrosative stress	Hydroxylamine reductase (EC 1.7.-.-)
		Nitrate and nitrite ammonification	Nitrite reductase probable electron transfer 4Fe-S subunit (EC 1.7.1.4)
			Assimilatory nitrate reductase large subunit (EC:1.7.99.4)
			Nitrite reductase probable [NAD(P)H] subunit (EC 1.7.1.4)
Dormancy and Sporulation		Spore Core Dehydration	Spore maturation protein B
			Spore maturation protein A

	Dormancy and Sporulation - no subcategory	Sporulation-associated proteins with broader functions	Protein of unknown function identified by role in sporulation (SpoVG)
			Peptidyl-tRNA hydrolase (EC 3.1.1.29)
			RNA polymerase sporulation specific sigma factor SigH
Respiration	Electron accepting reactions	Anaerobic respiratory reductases	Anaerobic sulfite reductase subunit C (EC 1.8.1.-)
			Anaerobic sulfite reductase subunit A
			Arsenate reductase (EC 1.20.4.1)
			Anaerobic sulfite reductase subunit B
			Anaerobic dehydrogenases, typically selenocysteine-containing
		Na(+)-translocating NADH-quinone oxidoreductase and rnf-like group of electron transport complexes	Electron transport complex protein RnfE
			Electron transport complex protein RnfA
			Electron transport complex protein RnfG
			Electron transport complex protein RnfD
			Electron transport complex protein RnfB
			Electron transport complex protein RnfC
		Respiratory dehydrogenases 1	Glycerol dehydrogenase (EC 1.1.1.6)
			NADH dehydrogenase (EC 1.6.99.3)
			Glycerol-3-phosphate dehydrogenase (EC 1.1.5.3)
			L-lactate dehydrogenase (EC 1.1.2.3)
		Succinate dehydrogenase	Fumarate reductase flavoprotein subunit (EC 1.3.99.1)
	Respiration - no subcategory	Biogenesis of c-type cytochromes	Cytochrome c-type biogenesis protein ResA
			Cytochrome c-type biogenesis protein CcdA (DsbD analog)
		Soluble cytochromes and functionally related electron carriers	Ferredoxin
Stress Response	Osmotic stress	Osmoregulation	Aquaporin Z
			Glycerol uptake facilitator protein
		Choline and Betaine Uptake and Betaine Biosynthesis	L-proline glycine betaine ABC transport system permease protein ProV (TC 3.A.1.12.1)
			Glycine betaine transporter OpuD
			Sarcosine oxidase alpha subunit (EC 1.5.3.1)
			L-proline glycine betaine binding ABC transporter protein ProX (TC 3.A.1.12.1)
	Oxidative stress	Oxidative stress	Rubrerythrin
			Ferric uptake regulation protein
			transcriptional regulator, Crp/Fnr family
			Superoxide reductase (EC 1.15.1.2)
			Fe2+/Zn2+ uptake regulation proteins
			Peroxide stress regulator
			Redox-sensitive transcriptional regulator (AT-rich DNA-binding protein)
		Glutathione: Biosynthesis and gamma-glutamyl cycle	Glutathione biosynthesis bifunctional protein gshF (EC 6.3.2.2)(EC 6.3.2.3)
		Glutathione: Non-redox reactions	Lactoylglutathione lyase (EC 4.4.1.5)
			Hydroxyacylglutathione hydrolase (EC 3.1.2.6)
		Rubrerythrin	Rubrerythrin
			Rubredoxin
			Superoxide reductase (EC 1.15.1.2)
		Redox-dependent regulation of nucleus processes	Nicotinate phosphoribosyltransferase (EC 2.4.2.11)
			Nicotinamidase (EC 3.5.1.19)
			NAD-dependent protein deacetylase of SIR2 family
			NAD-dependent glyceraldehyde-3-phosphate dehydrogenase (EC 1.2.1.12)
		Glutathione: Redox cycle	Glutaredoxin

		Glutaredoxins	Glutaredoxin
	Cold shock	Cold shock, CspA family of proteins	Cold shock protein CspA
	Heat shock	Heat shock dnaK gene cluster extended	Hypothetical radical SAM family enzyme, NOT coproporphyrinogen III oxidase, oxygen-independent
			Hypothetical radical SAM family enzyme in heat shock gene cluster, similarity with CPO of BS HemN-type
			Heat-inducible transcription repressor HrcA
			Chaperone protein DnaK
			tRNA-t(6)A37 methylthiotransferase
			Chaperone protein DnaJ
			Ribosomal RNA small subunit methyltransferase E (EC 2.1.1.-)
			tmRNA-binding protein SmpB
			Heat shock protein GrpE
			Translation elongation factor LepA
			Nucleoside 5-triphosphatase RdgB (dHAPTP, dITP, XTP-specific) (EC 3.6.1.15)
			Ribosomal protein L11 methyltransferase (EC 2.1.1.-)
			Signal peptidase-like protein
			FIG009886: phosphoesterase
			Ribonuclease PH (EC 2.7.7.56)
			rRNA small subunit methyltransferase I
	Stress Response - no subcategory	SigmaB stress responce regulation	Serine phosphatase RsbU, regulator of sigma subunit
		Hfl operon	GTP-binding protein HflX
		Carbon Starvation	Carbon starvation protein A
	Periplasmic Stress	Periplasmic Stress Response	Survival protein SurA precursor (Peptidyl-prolyl cis-trans isomerase SurA) (EC 5.2.1.8)
			Outer membrane protein H precursor
Metabolism of Aromatic Compounds	Peripheral pathways for catabolism of aromatic compounds	Quinate degradation	3-dehydroquinate dehydratase II (EC 4.2.1.10)
Amino Acids and Derivatives	Glutamine, glutamate, aspartate, asparagine; ammonia assimilation	Glutamine, Glutamate, Aspartate and Asparagine Biosynthesis	L-asparaginase I, cytoplasmic (EC 3.5.1.1)
			L-asparaginase (EC 3.5.1.1)
			Aspartate--ammonia ligase (EC 6.3.1.1)
			Aspartate aminotransferase (EC 2.6.1.1)
			Glutamate racemase (EC 5.1.1.3)
			Glutaminase (EC 3.5.1.2)
			Glutamate synthase [NADPH] small chain (EC 1.4.1.13)
			Glutamine synthetase type III, GlnN (EC 6.3.1.2)
			Aspartate ammonia-lyase (EC 4.3.1.1)
		Glutamine synthetases	Glutamine synthetase type III, GlnN (EC 6.3.1.2)
	Histidine Metabolism	Histidine Degradation	N-formylglutamate deformylase (EC 3.5.1.68)
			Formiminoglutamase (EC 3.5.3.8)
			Imidazolonepropionase (EC 3.5.2.7)
			Urocanate hydratase (EC 4.2.1.49)
			Histidine ammonia-lyase (EC 4.3.1.3)
			Glutamate formiminotransferase (EC 2.1.2.5)
	Arginine; urea cycle, polyamines	Urease subunits	Urease accessory protein UreD
			Urease accessory protein UreF
			Urease beta subunit (EC 3.5.1.5)
			Urease accessory protein UreG
			Urea channel UreI
			Urease gamma subunit (EC 3.5.1.5)
			Urease alpha subunit (EC 3.5.1.5)

	Polyamine Metabolism	Arginine/ornithine antiporter ArcD
		Spermidine Putrescine ABC transporter permease component potC (TC_3.A.1.11.1)
		Spermidine Putrescine ABC transporter permease component PotB (TC 3.A.1.11.1)
		Arginine decarboxylase, catabolic (EC 4.1.1.19)
		Putrescine transport ATP-binding protein PotA (TC 3.A.1.11.1)
		Agmatine deiminase (EC 3.5.3.12)
		Arginine decarboxylase (EC 4.1.1.19)
		Ornithine decarboxylase (EC 4.1.1.17)
		N-carbamoylputrescine amidase (3.5.1.53)
		5'-methylthioadenosine nucleosidase (EC 3.2.2.16)
		ABC transporter, periplasmic spermidine putrescine-binding protein PotD (TC 3.A.1.11.1)
	Arginine and Ornithine Degradation	Arginine/ornithine antiporter ArcD
		Arginine decarboxylase, catabolic (EC 4.1.1.19)
		Arginine decarboxylase (EC 4.1.1.19)
		Agmatine deiminase (EC 3.5.3.12)
		Ornithine decarboxylase (EC 4.1.1.17)
		Lysine-arginine-ornithine-binding periplasmic protein precursor (TC 3.A.1.3.1)
		N-carbamoylputrescine amidase (3.5.1.53)
	Urea decomposition	Urease accessory protein UreD
		Urease accessory protein UreF
		Urease beta subunit (EC 3.5.1.5)
		Urease accessory protein UreG
		Urea channel UreI
		Urease gamma subunit (EC 3.5.1.5)
		Urease alpha subunit (EC 3.5.1.5)
Lysine, threonine, methionine, and cysteine	Methionine Degradation	S-adenosylhomocysteine nucleosidase (EC 3.2.2.9)
		Pyruvate-flavodoxin oxidoreductase (EC 1.2.7.-)
		Methionine ABC transporter substrate-binding protein
		Methionine ABC transporter ATP-binding protein
		Methionine ABC transporter permease protein
		S-adenosylmethionine synthetase (EC 2.5.1.6)
		Methionine transporter MetT
		Adenosylhomocysteinase (EC 3.3.1.1)
	Methionine Biosynthesis	Methionine ABC transporter ATP-binding protein
		Duplicated ATPase component MtsB of energizing module of methionine-regulated ECF transporter
		S-adenosylmethionine synthetase (EC 2.5.1.6)
		O-succinylhomoserine sulfhydrylase (EC 2.5.1.48)
		Serine acetyltransferase (EC 2.3.1.30)
		Homoserine O-succinyltransferase (EC 2.3.1.46)
		5-methyltetrahydrofolate--homocysteine methyltransferase (EC 2.1.1.13)
		O-acetylhomoserine sulfhydrylase (EC 2.5.1.49)
		Transmembrane component MtsC of energizing module of methionine-regulated ECF transporter
		5,10-methylenetetrahydrofolate reductase (EC 1.5.1.20)
		Homoserine dehydrogenase (EC 1.1.1.3)
		Methionine ABC transporter substrate-binding protein
		S-adenosylhomocysteine nucleosidase (EC 3.2.2.9)
		Methionine ABC transporter permease protein
		Methionine transporter MetT
		Adenosylhomocysteinase (EC 3.3.1.1)

		Substrate-specific component MtsA of methionine-regulated ECF transporter
	Threonine and Homoserine Biosynthesis	Homoserine dehydrogenase (EC 1.1.1.3)
		Aspartate-semialdehyde dehydrogenase (EC 1.2.1.11)
		Aspartate aminotransferase (EC 2.6.1.1)
		Aspartokinase (EC 2.7.2.4)
	Threonine degradation	Threonine dehydratase (EC 4.3.1.19)
	Lysine Biosynthesis DAP Pathway, GJO scratch	Diaminopimelate decarboxylase (EC 4.1.1.20)
		N-acetyl-L,L-diaminopimelate deacetylase (EC 3.5.1.47)
		Diaminopimelate epimerase (EC 5.1.1.7)
		Aspartate-semialdehyde dehydrogenase (EC 1.2.1.11)
		2,3,4,5-tetrahydropyridine-2,6-dicarboxylate N-acetyltransferase (EC 2.3.1.89)
		4-hydroxy-tetrahydrodipicolinate reductase (EC 1.17.1.8)
		Aspartokinase (EC 2.7.2.4)
		4-hydroxy-tetrahydrodipicolinate synthase (EC 4.3.3.7)
	Lysine degradation	Lysine decarboxylase (EC 4.1.1.18)
	Lysine Biosynthesis DAP Pathway	Diaminopimelate decarboxylase (EC 4.1.1.20)
		N-acetyl-L,L-diaminopimelate deacetylase (EC 3.5.1.47)
		Diaminopimelate epimerase (EC 5.1.1.7)
		Aspartate-semialdehyde dehydrogenase (EC 1.2.1.11)
		2,3,4,5-tetrahydropyridine-2,6-dicarboxylate N-acetyltransferase (EC 2.3.1.89)
		4-hydroxy-tetrahydrodipicolinate reductase (EC 1.17.1.8)
		Aspartokinase (EC 2.7.2.4)
		4-hydroxy-tetrahydrodipicolinate synthase (EC 4.3.3.7)
Amino Acids and Derivatives - no subcategory	Creatine and Creatinine Degradation	Cytosine permease
		Cytosine deaminase (EC 3.5.4.1)
Branched-chain amino acids	Branched-Chain Amino Acid Biosynthesis	Branched-chain amino acid aminotransferase (EC 2.6.1.42)
		Threonine dehydratase (EC 4.3.1.19)
		Ketol-acid reductoisomerase (EC 1.1.1.86)
		Dihydroxy-acid dehydratase (EC 4.2.1.9)
		Acetolactate synthase large subunit (EC 2.2.1.6)
		Acetolactate synthase small subunit (EC 2.2.1.6)
Aromatic amino acids and derivatives	Common Pathway For Synthesis of Aromatic Compounds (DAHP synthase to chorismate)	Shikimate kinase I (EC 2.7.1.71)
		5-Enolpyruvylshikimate-3-phosphate synthase (EC 2.5.1.19)
		Shikimate 5-dehydrogenase I alpha (EC 1.1.1.25)
		3-dehydroquinate dehydratase II (EC 4.2.1.10)
		2-keto-3-deoxy-D-arabino-heptulosonate-7-phosphate synthase I beta (EC 2.5.1.54)
		3-dehydroquinate synthase (EC 4.2.3.4)
		Chorismate synthase (EC 4.2.3.5)
	Chorismate Synthesis	Shikimate kinase I (EC 2.7.1.71)
		5-Enolpyruvylshikimate-3-phosphate synthase (EC 2.5.1.19)
		Shikimate 5-dehydrogenase I alpha (EC 1.1.1.25)
		3-dehydroquinate dehydratase II (EC 4.2.1.10)
		2-keto-3-deoxy-D-arabino-heptulosonate-7-phosphate synthase I beta (EC 2.5.1.54)
		3-dehydroquinate synthase (EC 4.2.3.4)
		Chorismate synthase (EC 4.2.3.5)
	Chorismate: Intermediate for synthesis of Tryptophan, PAPA antibiotics, PABA, 3-hydroxyanthranilate and more.	Para-aminobenzoate synthase, amidotransferase component (EC 2.6.1.85)
		Isochorismatase (EC 3.3.2.1)

		Phenylalanine and Tyrosine Branches from Chorismate	Cyclohexadienyl dehydratase (EC 4.2.1.51)(EC 4.2.1.91)
			Biosynthetic Aromatic amino acid aminotransferase alpha (EC 2.6.1.57)
	Proline and 4-hydroxyproline	Proline Synthesis	Pyrroline-5-carboxylate reductase (EC 1.5.1.2)
		A Hypothetical Protein Related to Proline Metabolism	Hypothetical protein YggS, proline synthase co-transcribed bacterial homolog PROSC
			Pyrroline-5-carboxylate reductase (EC 1.5.1.2)
	Alanine, serine, and glycine	Glycine Biosynthesis	Serine hydroxymethyltransferase (EC 2.1.2.1)
		Alanine biosynthesis	Branched-chain amino acid aminotransferase (EC 2.6.1.42)
			Cysteine desulfurase (EC 2.8.1.7)
			Alanine racemase (EC 5.1.1.1)
		Serine Biosynthesis	Phosphoserine phosphatase (EC 3.1.3.3)
			Serine hydroxymethyltransferase (EC 2.1.2.1)
Sulfur Metabolism	Sulfur Metabolism - no subcategory	Thioredoxin-disulfide reductase	Thiol peroxidase, Tpx-type (EC 1.11.1.15)
			Alkyl hydroperoxide reductase protein F (EC 1.6.4.-)
			Alkyl hydroperoxide reductase protein C (EC 1.6.4.-)
			Thioredoxin reductase (EC 1.8.1.9)
		Galactosylceramide and Sulfatide metabolism	Alpha-galactosidase (EC 3.2.1.22)
			Beta-galactosidase (EC 3.2.1.23)
			probable beta-D-galactosidase
Phosphorus Metabolism	Phosphorus Metabolism - no subcategory	High affinity phosphate transporter and control of PHO regulon	Phosphate regulon transcriptional regulatory protein PhoB (SphR)
			Phosphate transport system permease protein PstC (TC 3.A.1.7.1)
			Phosphate transport ATP-binding protein PstB (TC 3.A.1.7.1)
			Phosphate transport system permease protein PstA (TC 3.A.1.7.1)
			Phosphate transport system regulatory protein PhoU
			Phosphate ABC transporter, periplasmic phosphate-binding protein PstS (TC 3.A.1.7.1)
			Polyphosphate kinase (EC 2.7.4.1)
			Phosphate regulon sensor protein PhoR (SphS) (EC 2.7.13.3)
		Phosphate metabolism	Phosphate regulon transcriptional regulatory protein PhoB (SphR)
			Phosphate transport system permease protein PstC (TC 3.A.1.7.1)
			Phosphate transport ATP-binding protein PstB (TC 3.A.1.7.1)
			Manganese-dependent inorganic pyrophosphatase (EC 3.6.1.1)
			Phosphate transport system permease protein PstA (TC 3.A.1.7.1)
			Exopolyphosphatase (EC 3.6.1.11)
			Phosphate transport system regulatory protein PhoU
			Predicted ATPase related to phosphate starvation-inducible protein PhoH
			Phosphate ABC transporter, periplasmic phosphate-binding protein PstS (TC 3.A.1.7.1)
			Alkaline phosphatase (EC 3.1.3.1)
			Alkaline phosphatase like protein
			Polyphosphate kinase (EC 2.7.4.1)
			Sodium-dependent phosphate transporter
			Phosphate regulon sensor protein PhoR (SphS) (EC 2.7.13.3)
		Polyphosphate	Polyphosphate kinase (EC 2.7.4.1)
			Exopolyphosphatase (EC 3.6.1.11)
Carbohydrates	Central carbohydrate metabolism	Methylglyoxal Metabolism	Methylglyoxal synthase (EC 4.2.3.3)
			Lactoylglutathione lyase (EC 4.4.1.5)
			Aldehyde dehydrogenase (EC 1.2.1.3)
			Hydroxyacylglutathione hydrolase (EC 3.1.2.6)
		Pyruvate:ferredoxin oxidoreductase	Pyruvate:ferredoxin oxidoreductase, delta subunit (EC 1.2.7.1)
			Pyruvate:ferredoxin oxidoreductase, alpha subunit (EC 1.2.7.1)
			Pyruvate-flavodoxin oxidoreductase (EC 1.2.7.-)
			Pyruvate:ferredoxin oxidoreductase, beta subunit (EC 1.2.7.1)
			Pyruvate:ferredoxin oxidoreductase, gamma subunit (EC 1.2.7.1)

	Pyruvate metabolism II: acetyl-CoA, acetogenesis from pyruvate	Aldehyde dehydrogenase (EC 1.2.1.3)
		Acetaldehyde dehydrogenase (EC 1.2.1.10)
		Phosphate acetyltransferase (EC 2.3.1.8)
		Acetate kinase (EC 2.7.2.1)
		NAD-dependent protein deacetylase of SIR2 family
		Acylphosphate phosphohydrolase (EC 3.6.1.7), putative
	Pyruvate Alanine Serine Interconversions	Branched-chain amino acid aminotransferase (EC 2.6.1.42)
		L-serine dehydratase (EC 4.3.1.17)
		Alanine racemase (EC 5.1.1.1)
	Dihydroxyacetone kinases	Phosphoenolpyruvate-dihydroxyacetone phosphotransferase (EC 2.7.1.121), subunit DhaM
		DHA-specific IIA component
		Phosphoenolpyruvate-dihydroxyacetone phosphotransferase (EC 2.7.1.121), ADP-binding subunit DhaL
		Phosphoenolpyruvate-dihydroxyacetone phosphotransferase (EC 2.7.1.121), dihydroxyacetone binding subunit DhaK
	Glycolysis and Gluconeogenesis	6-phosphofructokinase (EC 2.7.1.11)
		Triosephosphate isomerase (EC 5.3.1.1)
		Fructose-1,6-bisphosphatase, GlpX type (EC 3.1.3.11)
		Fructose-bisphosphate aldolase class II (EC 4.1.2.13)
		2,3-bisphosphoglycerate-independent phosphoglycerate mutase (EC 5.4.2.1)
		Glucose-6-phosphate isomerase (EC 5.3.1.9)
		Pyruvate kinase (EC 2.7.1.40)
		Enolase (EC 4.2.1.11)
		NAD-dependent glyceraldehyde-3-phosphate dehydrogenase (EC 1.2.1.12)
		Phosphoglycerate kinase (EC 2.7.2.3)
	Pentose phosphate pathway	Transketolase, N-terminal section (EC 2.2.1.1)
		Ribose-phosphate pyrophosphokinase (EC 2.7.6.1)
		6-phosphogluconolactonase (EC 3.1.1.31), eukaryotic type
		Transaldolase (EC 2.2.1.2)
		Ribose 5-phosphate isomerase B (EC 5.3.1.6)
		Transketolase, C-terminal section (EC 2.2.1.1)
		Ribulose-phosphate 3-epimerase (EC 5.1.3.1)
	Pyruvate metabolism I: anaplerotic reactions, PEP	Oxaloacetate decarboxylase beta chain (EC 4.1.1.3)
		Phosphoenolpyruvate carboxykinase [ATP] (EC 4.1.1.49)
		NADP-dependent malic enzyme (EC 1.1.1.40)
		Pyruvate kinase (EC 2.7.1.40)
		Malate permease
Aminosugars	Chitin and N-acetylglucosamine utilization	PTS system, N-acetylglucosamine-specific IIB component (EC 2.7.1.69)
		Glucosamine-6-phosphate deaminase (EC 3.5.99.6)
		N-Acetyl-D-glucosamine ABC transport system, permease protein 2
		Chitinase (EC 3.2.1.14)
		N-acetylglucosamine-6-phosphate deacetylase (EC 3.5.1.25)
Di- and oligosaccharides	Sucrose utilization	Sucrose operon repressor ScrR, LacI family
		PTS system, sucrose-specific IIC component (EC 2.7.1.69)
		PTS system, sucrose-specific IIB component (EC 2.7.1.69)
		Fructokinase (EC 2.7.1.4)
	Maltose and Maltodextrin Utilization	4-alpha-glucanotransferase (amylomaltase) (EC 2.4.1.25)
		Maltose/maltodextrin ABC transporter, permease protein MalF
		Maltose-6'-phosphate glucosidase (EC 3.2.1.122)
		Neopullulanase (EC 3.2.1.135)
		Glycogen phosphorylase (EC 2.4.1.1)
		Alpha-glucosidase (EC 3.2.1.20)
		Aldose 1-epimerase (EC 5.1.3.3)

		PTS system, maltose and glucose-specific IIC component (EC 2.7.1.69)
		Multiple sugar ABC transporter, ATP-binding protein
		Maltose/maltodextrin transport ATP-binding protein MalK (EC 3.6.3.19)
		PTS system, maltose and glucose-specific IIB component (EC 2.7.1.69)
		Maltoporin (maltose/maltodextrin high-affinity receptor, phage lambda receptor protein)
		Maltose/maltodextrin ABC transporter, permease protein MalG
	Beta-Glucoside Metabolism	PTS system, cellobiose-specific IIB component (EC 2.7.1.69)
		Outer surface protein of unknown function, cellobiose operon
		6-phospho-beta-glucosidase (EC 3.2.1.86)
		PTS system, cellobiose-specific IIC component (EC 2.7.1.69)
		PTS system, cellobiose-specific IIA component (EC 2.7.1.69)
		Beta-glucosidase (EC 3.2.1.21)
	Lactose and Galactose Uptake and Utilization	UDP-glucose 4-epimerase (EC 5.1.3.2)
		Galactose/methyl galactoside ABC transport system, permease protein MglC (TC 3.A.1.2.3)
		Alpha-galactosidase (EC 3.2.1.22)
		Galactokinase (EC 2.7.1.6)
		Aldose 1-epimerase (EC 5.1.3.3)
		Galactose/methyl galactoside ABC transport system, D-galactose-binding periplasmic protein MglB (TC 3.A.1.2.3)
		Beta-galactosidase (EC 3.2.1.23)
		Galactose/methyl galactoside ABC transport system, ATP-binding protein MglA (EC 3.6.3.17)
		Galactose-1-phosphate uridylyltransferase (EC 2.7.7.10)
	Lactose utilization	Beta-galactosidase (EC 3.2.1.23)
		Evolved beta-D-galactosidase, alpha subunit
		Evolved beta-D-galactosidase transcriptional repressor
		Galactoside O-acetyltransferase (EC 2.3.1.18)
One-carbon Metabolism	One-carbon metabolism by tetrahydropterines	Formate--tetrahydrofolate ligase (EC 6.3.4.3)
		Formiminotetrahydrofolate cyclodeaminase (EC 4.3.1.4)
		Methylenetetrahydrofolate dehydrogenase (NADP+) (EC 1.5.1.5)
		5,10-methylenetetrahydrofolate reductase (EC 1.5.1.20)
		Methenyltetrahydrofolate cyclohydrolase (EC 3.5.4.9)
Organic acids	Glycerate metabolism	2-hydroxy-3-oxopropionate reductase (EC 1.1.1.60)
		D-glycerate transporter (predicted)
		Pyruvate kinase (EC 2.7.1.40)
		Glycerate kinase (EC 2.7.1.31)
	Lactate utilization	L-lactate dehydrogenase (EC 1.1.2.3)
	Citrate Metabolism, Transport, and Regulation	Na(+)Citrate OH(-) antiporter
		Citrate lyase gamma chain, acyl carrier protein (EC 4.1.3.6)
		[Citrate [pro-3S]-lyase] ligase (EC 6.2.1.22)
		Citrate lyase beta chain (EC 4.1.3.6)
		Citrate lyase alpha chain (EC 4.1.3.6)
Fermentation	Fermentations: Mixed acid	D-lactate dehydrogenase (EC 1.1.1.28)
		Alcohol dehydrogenase (EC 1.1.1.1)
		Acetaldehyde dehydrogenase (EC 1.2.1.10)
		Formate efflux transporter (TC 2.A.44 family)
		Pyruvate formate-lyase activating enzyme (EC 1.97.1.4)
		L-lactate dehydrogenase (EC 1.1.1.27)
		Sugar/maltose fermentation stimulation protein homolog
		Pyruvate formate-lyase (EC 2.3.1.54)
		Acetate kinase (EC 2.7.2.1)
		Phosphate acetyltransferase (EC 2.3.1.8)
		Acetolactate synthase large subunit (EC 2.2.1.6)

	Acetolactate synthase subunits	Acetolactate synthase small subunit (EC 2.2.1.6)
	Fermentations: Lactate	D-lactate dehydrogenase (EC 1.1.1.28)
		L-lactate dehydrogenase (EC 1.1.1.27)
		Acetaldehyde dehydrogenase (EC 1.2.1.10)
		Phosphate acetyltransferase (EC 2.3.1.8)
		Acetate kinase (EC 2.7.2.1)
Sugar alcohols	Glycerol and Glycerol-3-phosphate Uptake and Utilization	NADH peroxidase (EC 1.11.1.1)
		Glycerol-3-phosphate ABC transporter, permease protein UgpE (TC 3.A.1.1.3)
		Glycerol-3-phosphate dehydrogenase (EC 1.1.5.3)
		Glycerol-3-phosphate dehydrogenase [NAD(P)+] (EC 1.1.1.94)
		Glycerol-3-phosphate ABC transporter, permease protein UgpA (TC 3.A.1.1.3)
		Glycerol kinase (EC 2.7.1.30)
		Glycerol-3-phosphate ABC transporter, periplasmic glycerol-3-phosphate-binding protein (TC 3.A.1.1.3)
		Glycerophosphoryl diester phosphodiesterase (EC 3.1.4.46)
		Glycerol uptake facilitator protein
Polysaccharides	Glycogen metabolism	Glucose-1-phosphate adenylyltransferase (EC 2.7.7.27)
		4-alpha-glucanotransferase (amylomaltase) (EC 2.4.1.25)
		1,4-alpha-glucan (glycogen) branching enzyme, GH-13-type (EC 2.4.1.18)
		Glycogen biosynthesis protein GlgD, glucose-1-phosphate adenylyltransferase family
		Glycogen phosphorylase (EC 2.4.1.1)
		Glycogen debranching enzyme (EC 3.2.1.-)
		Glycogen synthase, ADP-glucose transglucosylase (EC 2.4.1.21)
Monosaccharides	Mannose Metabolism	Alpha-mannosidase (EC 3.2.1.24)
		Mannose-6-phosphate isomerase (EC 5.3.1.8)
		Mannose-1-phosphate guanylyltransferase (GDP) (EC 2.7.7.22)
	D-ribose utilization	Ribose ABC transport system, ATP-binding protein RbsA (TC 3.A.1.2.1)
		Ribokinase (EC 2.7.1.15)
		Ribose 5-phosphate isomerase B (EC 5.3.1.6)
	Deoxyribose and Deoxynucleoside Catabolism	Purine nucleoside phosphorylase (EC 2.4.2.1)
		Ribokinase (EC 2.7.1.15)
		Phosphopentomutase (EC 5.4.2.7)
		Pyrimidine-nucleoside phosphorylase (EC 2.4.2.2)
		Deoxyribose-phosphate aldolase (EC 4.1.2.4)
	L-ascorbate utilization (and related gene clusters)	L-ribulose-5-phosphate 4-epimerase (EC 5.1.3.4)
		3-keto-L-gulonate 6-phosphate decarboxylase
		L-xylulose 5-phosphate 3-epimerase (EC 5.1.3.-)
		Probable L-ascorbate-6-phosphate lactonase UlaG (EC 3.1.1.-) (L-ascorbate utilization protein G)
		Ascorbate-specific PTS system, EIIC component
	D-galactarate, D-glucarate and D-glycerate catabolism	2-hydroxy-3-oxopropionate reductase (EC 1.1.1.60)
		D-glycerate transporter (predicted)
		Glycerate kinase (EC 2.7.1.31)
	Fructose utilization	Phosphoenolpyruvate-protein phosphotransferase of PTS system (EC 2.7.3.9)
		PTS system, fructose-specific IIC component (EC 2.7.1.69)
		Transcriptional repressor of the fructose operon, DeoR family
		PTS system, fructose-specific IIB component (EC 2.7.1.69)
		Transaldolase (EC 2.2.1.2)
		PTS system, fructose-specific IIA component (EC 2.7.1.69)
		Fructokinase (EC 2.7.1.4)
		2-hydroxy-3-oxopropionate reductase (EC 1.1.1.60)

	D-galactarate, D-glucarate and D-glycerate catabolism - gjo	D-glycerate transporter (predicted)
		Glycerate kinase (EC 2.7.1.31)
